# Experiences of Immersive Virtual Reality Among Older Adults With Physical Disabilities: Qualitative Process Evaluation of a Multicenter Randomized Controlled Trial

**DOI:** 10.2196/101815

**Published:** 2026-10-29

**Authors:** Jing Jing Su, Rick Yiu Cho Kwan, Janet Lok Chun Lee, Fowie Ng, Sally Wai Chi Chan

**Affiliations:** 1 School of Nursing Tung Wah College Hong Kong China (Hong Kong); 2 Department of Rehabilitation Sciences Hong Kong Polytechnic University Hong Kong China (Hong Kong); 3 School of Management Tung Wah College Hong Kong China (Hong Kong); 4 Tung Wah College Hong Kong China (Hong Kong)

**Keywords:** Immersive Virtual Reality Therapeutic Programme, immersive virtual reality, IVR, IVRTP, older adults, physical disabilities, qualitative, randomized controlled trial, RCT, reminiscence, tourism

## Abstract

**Background:**

Emerging evidence indicates that immersive virtual reality can be used therapeutically and has demonstrated efficacy in promoting the mental health of older adults. Nevertheless, there remains a paucity of qualitative research examining the specific experiences underpinning these benefits.

**Objective:**

This study aimed to explore the perspectives of older adults who received the 12-session Immersive Virtual Reality Therapeutic Programme (IVRTP) intervention and health-related undergraduate students who delivered it as interventionists, within the context of residential care homes.

**Methods:**

A descriptive qualitative study was conducted as a qualitative exploration embedded in a multicenter randomized controlled trial evaluating the effects of IVRTP in promoting the mental health of older adults with disabilities living in residential homes. The IVRTP incorporated therapeutic elements of tourism, natural scenes, and reminiscence. Data were collected through focus group interviews and analyzed using thematic analysis.

**Results:**

Seven groups of older adults (n=28) and 3 groups of student interventionists (n=9) participated in the study. Five themes emerged: IVRTP contributes to “broadening horizons” through embracing new technology, learning new things, and engaging in new activities. Participants noted that the IVRTP provided “a realistic journey and immersive escape” from their residential home environment and mobility issues. They expressed that the IVRTP evoked “reminiscent memories” from the past and were amazed at the observed changes in tourist destinations over time. The interviews were filled with laughter and affirmations of positive emotions associated with this “joyful adventure,” although a few participants mentioned “minor discomfort,” such as eye soreness and dizziness, from wearing the head-mounted devices. Students’ accounts corroborated these themes; their meaning-making and colearning with older adults underscored the pedagogical value of authentic, community-based, intergenerational learning and reinforced the case for integrating a community practicum into the curriculum.

**Conclusions:**

Implementing an IVRTP in residential care settings, particularly for older adults with mobility limitations, is positively viewed as an accessible, enjoyable, and beneficial intervention to support psychosocial well-being. Therapeutically oriented content delivered through a structured protocol, coupled with the use of advanced IVR technology, was appreciated by both older adults and student interventionists.

## Introduction

Physical disabilities are consistently associated with higher levels of depression in late life [1]. This concern is particularly salient for older adults with physical disabilities residing in residential care settings, who often face reduced freedom, limited independence, restrictions on various activities and life experiences, and social isolation [2]. They tend to exhibit greater psychological distress and reduced energy and vitality than their community-dwelling peers [3]. Optimizing strategies to allow exposure to new experiences with therapeutic components may help reduce psychological distress and the ensemble of associated negative effects.

In response, immersive virtual reality (IVR) has expanded rapidly as an adjunctive therapeutic tool in mental health care for older adults with physical disabilities [4]. Immersive technologies refer to the capability to deliver inclusive, surrounding, vivid sensory displays, and virtual reality broadly refers to computer-generated interactive simulation that creates a subjective sense of presence in the virtual environment. Immersion levels vary: semi-IVR generally uses large screens to create simulation experiences while keeping users connected to the real world, whereas IVR blocks out the real world using devices such as head-mounted displays and controllers [5]. Existing IVR-based interventions encompass motor-cognitive training, virtual gardening, outdoor leisure activities, virtual tourism, reminiscence-based interventions, and objective assessment of motor and cognitive performance during ecologically valid activities [6-10]. IVR can deliver personalized psychological interventions by tailoring reminiscence content [11] and enabling exposure to environments and scenarios that provide safe, positive experiences, thereby helping individuals question the thoughts that sustain paranoid beliefs [12]. Recent reviews and large-scale randomized controlled trials (RCTs) found that IVR-based interventions are effective in improving mental health among older adults living in the community as well as in residential facilities [13]. A systematic review of IVR interventions for reminiscence therapy found that they could enhance psychological well-being and cognitive outcomes [14]. Another recent meta-analysis of diverse IVR-based interventions, including gardening, cognitive and motor training, and exercise games, found that these interventions significantly improved depression, anxiety, quality of life, emotional well-being, and stress among older adults with chronic conditions who live in communities as well as in residential facilities [15]. Such therapeutic programs are intrinsically valuable for this cohort because they provide opportunities to escape the constraints of daily life in care settings, transporting participants to stimulating, calming, and enriching environments despite limitations in mobility, energy, and activity participation [16].

Despite growing evidence supporting the implementation and effectiveness of IVR-based interventions, large-scale trials and routine implementation encounter substantial barriers at both participant and health care provider levels. For older adults, key concerns include acceptability, cybersickness, and other adverse events; limited accessibility for individuals with cognitive, sensory, or mobility impairments; and low digital literacy. For health care professionals and organizations, challenges include requirements for staff training and supervision, as well as the costs of program development, hardware procurement and maintenance, patient monitoring, and ongoing technical support, all of which can affect adoption, adherence, fidelity, safety, and clinical effectiveness [17,18].

In recent years, patient and public involvement and engagement (PPIE) has been increasingly advocated to help bridge the evidence-practice gap by involving stakeholders throughout the research and implementation process [19]. PPIE fosters a comprehensive understanding of experiences related to both participation and the professionals who participated in or delivered the intervention, which is essential for improving the acceptability, appropriateness, effectiveness, and sustainability of interventions. Using qualitative process evaluation to explore IVR intervention use and real-world influence aligns well with PPIE initiatives. In-depth qualitative analysis of older adults’ experiences can capture user variability in how they interact with the IVR program and thus expand the explanation of objective usage patterns (eg, times of usage) and/or reveal critical aspects undetected by the quantitative usage data (eg, immersion in the simulated world) [20]. More importantly, qualitative data can capture health-related cognitions and offline influences within broader social and contextual settings, such as interpersonal conversations arising after IVR participation. For older adults, PPIE can elucidate perceived mechanisms of benefit and inform the design and delivery of IVR interventions, for example, content, interface, session duration, and support needs, to enhance satisfaction, engagement, and adherence. For interventionists, delivery methods can substantially influence outcomes; therefore, incorporating their experiences helps identify workflow constraints, training requirements, and fidelity considerations.

Several qualitative investigations have explored older adults’ experiences, perceptions, acceptability, and facilitators regarding IVR-based interventions. A recent qualitative systematic synthesis supported older adults’ acceptability of IVR, indicating that they generally respond positively to IVR and are willing to tolerate IVR-related discomfort to enjoy its benefits [17]. Many perceived IVR as a platform that facilitates access to activities and environments that may be less accessible in the real world [17], though some expressed a preference for in-person, real-world activities [21]. A review also documented that age-related sensorimotor and cognitive decline affect immersive virtual reality use [22]. Although some evidence exists regarding the general subjective experiences of older adults and health care professionals, most of these studies have been limited to pilot feasibility designs [23], and the perspectives of these groups have typically been collected separately. There is therefore a lack of IVR research that incorporates process evaluation data from multiple involved stakeholders, which is necessary to generate direct implementation insights for future real-world practice. Moreover, understanding remains limited regarding how older adults with physical disabilities experience IVR, as previous studies have largely focused on the general older adult population. There is also insufficient knowledge of how intervention providers perceive IVR, particularly given that a substantial proportion of older adults experience technical difficulties when using IVR. In one study, a nature-based IVR intervention comprising 5 natural scenes (45 seconds to 3 minutes each) was delivered to older adults with cognitive and/or physical impairments, followed by qualitative interviews. Findings supported the program’s tolerability, safety, and transient enjoyment [16]; however, its one-off nature and lack of structure as a therapeutic program precluded deeper exploration of impact and implications.

In contrast, we developed a multidisciplinary Immersive Virtual Reality Therapeutic Programme (IVRTP) comprising 12 sessions delivered over 6 weeks, incorporating natural scenes, tourism, and reminiscence for older adults with physical disabilities [24], and evaluated its efficacy in a multicenter RCT that demonstrated significant improvements in quality of life [25]. Therefore, this study aimed to explore the perspectives of (1) older adults residing in residential homes who received the IVRTP and (2) health-related undergraduate students who delivered the IVRTP intervention as part of their practicum. Examining students’ perspectives alongside those of older adults provides insights into acceptability and feasibility for both groups and clarifies how educational institutions might augment the workforce required to deliver such programs while simultaneously meeting students’ training requirements.

## Methods

### Ethical Considerations

This study received ethical approval from the Research Ethics Committee of Tung Wah College, Hong Kong (REC2023158). The qualitative component was embedded within a multicenter RCT assessing the effect of IVRTP (ClinicalTrials.gov: NCT05818579) [25].

All participants provided written informed consent after the study was explained in full, and interviewers offered support to older adults as needed. Recognizing the potential vulnerability of older participants, interviews were conducted in comfortable settings and could be paused or discontinued if fatigue or distress arose. Confidentiality and anonymity were strictly safeguarded: data were stored securely, identifying details were not reported, and pseudonyms were used in discussions. No incentives were offered. All procedures adhered to core ethical principles, including respect for autonomy, protection from harm, and equitable treatment of both older adults and student interventionists.

### Study Design

A descriptive qualitative design was adopted, using semistructured focus group interviews and thematic analysis. This design enables spontaneous expression and responses, fostering active interaction among participants as they share experiences with the IVRTP [26,27]. The study presentation was guided by the 21-item SRQR (Standards for Reporting Qualitative Research) checklist [28] (Multimedia Appendix 1).

### Setting, Participants, and Sampling

This study presents the qualitative process evaluation embedded within a multicenter RCT evaluating the effects of IVRTP [25]. The main RCT was carried out in 37 long-term care facilities (LTCFs) in Hong Kong, after all residential care homes registered with the Social Welfare Department had been invited to participate in the main RCT through multiple channels, including postal mail, email, telephone, and instant messaging apps. These LTCFs provide residential accommodation, personal care, meals, basic medical and nursing support, and social activities for older adults with physical disabilities who are unable to live independently. Upon completion of the intervention, we invited the 37 LTCFs to refer participants who had participated in the IVRTP to attend the interview, and 7 facilities expressed interest in the qualitative process evaluation. Those that agreed to participate were enrolled as study centers, after which participant recruitment was initiated. Interviews were conducted over a 22-month period (May 2023-March 2025). Postintervention focus group interviews were conducted separately with older adults and student interventionists in quiet, private rooms within the LTCFs to facilitate open, undisturbed, and confidential discussion. Each focus group comprised 3 to 5 participants to maximize participation, ensure comfort, and facilitate in-depth discussion among a vulnerable population [29].

Participants included both older adults and student interventionists. Older adults were eligible if they were aged 60 years or older; resided in a long-term care home; lived with physical disability, operationalized as a modified Barthel Index (MBI) score of 90 or below indicating at least moderate dependence [30]; and had no severe memory or cognitive impairment that would impede meaningful participation in group discussions, as determined by the Hong Kong version of the Montreal Cognitive Assessment using a cutoff at the second percentile adjusted for age and education [31]. Eligible older adults also had to have completed the IVRTP with a participation rate exceeding 80% and be willing to share their experiences. Student interventionists were undergraduate students from Tung Wah College who had actively facilitated IVRTP sessions as part of their required community service.

Purposive sampling was used to recruit participants with diverse sociodemographic characteristics, different improvements in the primary outcome of mental well-being, and substantial engagement in IVRTP [26]. Recruitment was promoted through the participating LTCFs, and facilities that expressed willingness to support the study referred participants with diverse characteristics. To maximize sample variation, the research team reviewed the characteristics of referred participants, their IVRTP completion rates, and their outcome improvements, and then advised facilities on the types of participants to refer subsequently. Focus group sample size was determined using a saturation-based approach: code saturation was attained after 3 groups and meaning saturation after 6, with 1 additional older adult group conducted to confirm saturation [32]. Given the smaller number of student interventionists, interviews with students constituted a secondary implementation-related finding; saturation for the subgroup was limited to code saturation, which was achieved with 2 groups.

### Intervention: IVRTP

Intervention details are provided in the published protocol [24]. IVRTP content was codeveloped by a nurse-led multidisciplinary team, including nurses, gerontology specialists, digital technology experts, and older adults, to ensure theoretical grounding, cultural relevance, and usability. The app was developed by the research team in collaboration with a technology company using Unity and is available on SideQuest under the title TWC Elderly VR [33]. The intervention followed a structured 12-session protocol: 1-hour sessions delivered twice weekly over 6 weeks. Each session included 3 phases: a 10-minute briefing, a 30-minute IVR immersion, and a 10-minute guided group discussion, with phase durations adjusted as needed to accommodate participants’ needs. Participants who missed sessions could complete up to 2 targeted make-up sessions at the end of the program.

The program used Meta’s Oculus Quest 2 head-mounted displays with Touch controllers for interaction. A custom tablet-based monitoring application (Apple iPad, ninth generation) enabled facilitators to mirror participants’ views in real time, adjust settings, and promptly address technical issues or discomfort. Each functional IVRTP unit comprised a head-mounted display and controller, a tablet computer, software containing 12 therapeutic IVR experiences installed on the headset, and a control interface on the tablet. Participants used the headset and controller, while facilitators operated the tablet to guide and manage session delivery.

The IVRTP comprised 3 modules—natural scenes, tourism, and reminiscence. Natural scenes provided calm, fully interactive 3D environments (eg, beaches and forests) in which participants could explore and manipulate virtual objects. Tourism offered semi-interactive 360° video tours of Hong Kong landmarks (eg, Ngong Ping Village and Victoria Peak), allowing control of viewing angle and movement speed. Reminiscence recreated nostalgic Hong Kong settings (eg, Tai O Fisherman Village and Old Hong Kong Streets) as fully interactive 3D spaces designed to evoke autobiographical memories and promote social interaction. All 360° video content was delivered in 4K resolution (about 4096 pixels across its width).

The IVRTP was delivered in 3 phases. During briefing, the interventionist fitted the head-mounted display, connected the tablet to the headset, optimized system settings, provided instructions, and oriented participants to the virtual environment. During the IVR session, the interventionist guided participants in exploring and interacting with the virtual environment in real time (eg, collecting seashells on the beach). During discussion, the interventionist facilitated debriefing by inviting participants to share experiences and prompting group dialogue arising from the session.

Undergraduate students in nursing or allied health served as interventionists after completing a standardized 2-hour workshop on IVR operation, geriatric communication, and safety protocols. Training covered hardware setup, software use, troubleshooting, and risk management (including mitigation of IVR-induced sickness). Student interventionists were coached to communicate with older adults of varying abilities using clear instructions and active listening, and they practiced group facilitation through scenario-based role-plays. Care home nurses, supported by the project’s nurse researchers, provided on-site supervision, delivered additional training to interventionists and support staff, and offered operational guidance during implementation.

Sessions were conducted in small groups (2-3 participants per interventionist) to ensure individualized support. Multiplayer functionality enabled shared virtual experiences and promoted social interaction. Participants could use IVR while seated, including in beds or wheelchairs, in communal rooms or their own bedrooms according to their preference. Wireless headsets paired with tablet controls supported participation regardless of mobility or functional limitations.

### Data Collection

Semistructured focus group interviews were conducted to elicit participants’ perspectives while maintaining alignment with the study objectives [34]. Two tailored interview guides were developed specifically for this study, informed by the research team’s expertise and the extant literature, and are provided in Multimedia Appendix 2 (older adults’ version and student interventionists’ version). The developers had extensive experience in the design and implementation of therapeutic IVRTP and in qualitative interviewing. The guides comprised open-ended questions covering motivations for participation; overall experiences with IVRTP, including realism, immersion, technical performance, and social interaction; and perceived effects on mental well-being and social connectedness among older adults. Student interventionists were interviewed regarding their experiences delivering the intervention; motivations for participation; strategies to promote IVRTP use among older adults; and debriefing on learning experiences, such as communication skills and attitudes toward aging. Both older adults and student interventionists were invited to offer reflections on unexpected or memorable aspects and to provide recommendations for program improvement. No pilot interviews were conducted.

Two members of the project team, a research project manager and a research assistant, who were knowledgeable about the study but not directly involved in delivering the intervention or teaching and who were experienced in qualitative interviewing, conducted the interviews. Their subject-matter familiarity supported a nuanced understanding of both participant and interventionist perspectives, consistent with the view that interviewer-interviewee relationships operate along a continuum rather than a rigid insider/outsider dichotomy [35]. To preserve confidentiality and manage group dynamics, no identifying information (eg, participants’ names or educational background) was collected or disclosed during interviews [36,37]. Each interview lasted approximately 25 to 40 minutes and, with participants’ consent, was audio-recorded using the built-in voice recorder on an Apple iPhone to enable verbatim transcription. Interviews were conducted in Cantonese, the participants’ primary language, either immediately or shortly after completion of the IVRTP intervention cycles, to minimize recall bias and promote fresh reflection.

### Data Analysis

Thematic analysis was undertaken to identify recurring patterns in participants’ views, experiences, and practices across the dataset [38]. Thematic analysis is a systematic approach to qualitative data analysis that seeks to identify and interpret patterns across a dataset, thereby providing meaningful insights into participants’ IVRTP experiences and perspectives [39]. Several discrete steps are involved in thematic analysis, including familiarizing oneself with the data, generating initial codes, searching for themes, reviewing themes, and, finally, defining themes [38]. Audio recordings were transcribed verbatim and translated into English by the first author, who is bilingual in Cantonese and English; field notes were also compiled. The accuracy of the transcripts was verified by the interviewers through comparison with the original audio recordings. Using an inductive approach, the first author conducted line-by-line coding of the first 4 transcripts in English, assigning short descriptive labels to segments of text that captured their meaning and content [40]. The researcher then met with the interviewers to discuss these initial codes, ensuring that they represented participants’ viewpoints and balanced readability with authenticity [41]. These discussions informed the development of a preliminary codebook, which included code names, definitions, and illustrative quotations. Additional transcripts were then coded, with new codes added as needed and existing code definitions refined, resulting in iterative revision of the codebook by the first author. Following coding, all codes were reviewed across transcripts and compared constantly to explore patterns, variation, and relationships within and across participants’ accounts. Related codes were grouped into broader categories based on conceptual similarity and recurrence in the dataset, and these categories were then organized into subthemes and overarching themes. A deductive review was subsequently undertaken to assess whether the candidate themes adequately captured all coded data and whether any codes had been insufficiently represented or omitted. Candidate themes were reviewed against both the coded data and the full dataset to ensure that they accurately reflected participants’ accounts, were distinct from one another, and provided comprehensive coverage of the codes. Final themes were refined and agreed upon through extensive team discussions.

### Trustworthiness

Trustworthiness was addressed using Guba’s 4 criteria: credibility, transferability, dependability, and confirmability [42]. A rigorous approach to data collection and analysis was maintained throughout. Credibility was ensured through prolonged engagement, reflexivity, and triangulation. To support prolonged engagement, the focus group interviews were conducted by project staff who were familiar with the study protocol and implementation but were not involved in delivering the immersive IVRTP intervention or in training or teaching the student interventionists. This arrangement facilitated a nuanced understanding of both participant and interventionist perspectives while promoting interviewer impartiality and reducing social desirability bias. Reflexivity was addressed by managing researchers’ potential biases using a standardized interview guide with open-ended questions and probes, as well as by following a well-established thematic analysis approach. In addition, reflexivity was strengthened by explicitly acknowledging that the primary data analyst was a doctoral-trained nurse with extensive training and research experience in qualitative research, IVR-based interventions, and thematic analysis, and that the research team had a strong professional interest in IVR-based interventions. These relevant professional perspectives were considered throughout data collection and analysis to ensure that negative views and experiences of discomfort were not overlooked, thereby minimizing undue influence, such as overly favorable interpretations. Triangulation was achieved through the use of multiple data collection methods, including interviews, observations, and field notes [43].

Transferability was enhanced through thick description, maximal variation sampling, and a focus group interviewing approach in which group dynamics were actively facilitated to encourage equitable contribution. Detailed descriptions of the intervention components, participant eligibility criteria, and the context of intervention delivery allow readers to assess the extent to which the study context resembles their own and to judge the applicability of the findings to other settings. To maximize sample variation and enrich the data, participants were purposively recruited from among those who completed at least 80% of the IVRTP and who represented different socioeconomic backgrounds. Group dynamics were actively managed to promote equitable participation and authentic dialogue. Interviewers encouraged participant-to-participant interaction to foster collective reflection rather than interviewer-led exchanges. Dominant contributors were respectfully redirected, and quieter participants were explicitly invited to speak; small group sizes further supported balanced engagement. To mitigate groupthink and elicit diverse perspectives, interviewers probed for differing or dissenting views and discouraged premature consensus. Nonverbal cues and interactional dynamics were noted during sessions and considered during subsequent analysis [44].

Dependability was ensured through detailed documentation and reporting of each step of the research process, thereby enhancing transparency and enabling others to understand and potentially replicate the study. An audit trail was maintained throughout the research process to ensure traceability, with documentation of the raw data, methodological decisions, including study sites and sampling and recruitment strategies, as well as coding procedures, analytic processes, and process notes [43].

Confirmability was ensured through peer debriefing and reflective journaling. For peer debriefing, the first author held debriefing meetings with the interviewers to ensure that the codes accurately reflected the participants’ quotations. Reflective journaling allowed the researchers to document their evolving thoughts, assumptions, biases, and reflections throughout the study, thereby supporting a more balanced and transparent interpretation of the data. Member checking was not conducted to minimize the burden on participants, particularly because their perspectives were intended to inform experiential understanding rather than to support the co-design of the intervention [45].

## Results

### Participants’ Characteristics

The study interviewed 7 groups of older adults (n=28), most of whom were female (20/28, 71.42%). The mean years of education were 6.97 (SD 4.78) years, the mean length of stay at the facility was 3.62 (SD 5.62) years, and the mean MBI score was 73.45 (SD 14.21). Additionally, 3 groups of interventionists (n=9), comprising students in nursing (5/9, 55.56%) and allied health, were interviewed; more than half were female (5/9, 55.56%).

### Broadening Horizons

Older adults expressed curiosity about advanced technology, a desire to participate in novel activities and acquire new knowledge, and appreciation of learning opportunities afforded by technological platforms. They used various expressions to articulate how these experiences broadened their horizons, such as “my eyes going on trip,” “learn high-end things,” and “magic glasses (goggles).” Many participants highlighted a lack of exposure to novel experiences while residing in residential homes, especially noting the limited availability of outdoor activities, as their time was predominantly spent indoors watching television or playing online games like MahJong and Sudoku. Through the IVRTP intervention, they found themselves surprised by the opportunity to revisit familiar places and learn about new cities, natural environments, plants, and animals without payment.

*Through this IVR experience, we’ve really broadened our horizons.It feels like getting to travel for free, like your eyes are going on a trip.* [Group 3, number 1]

What I learn is that science and tech these days are changing super-fast. Us older folks can’t really keep up at all. But just by attending this, we can see that the world is constantly advancing in ways we can’t even imagine.

Group 3, number 4

The student interventionists reinforced this narrative by stating that older adults found IVR devices novel and interesting. They exhibited hesitation at the beginning, but after receiving instructions from student interventionists, they were excited to learn new things. They shared that these older adults frequently miss out on such enriching experiences due to mobility challenges; however, IVR has the potential to bring outdoor experiences directly to them. Some students highlighted that interventions such as this are in high demand—people are generally curious about documentaries about wildlife migration, Arctic penguins, or volcanic eruptions, and such experiences should be open to everyone who shows interest.

They got to “visit” the Antarctic and see penguins through this program. It made me realize that, at least for those interested in broadening their horizons, this program can help them see things they’ve always wanted to, even if their physical abilities or unfamiliarity with technology might have been barriers otherwise. Instead of just watching the news all day, they get to explore the world virtually.

Student interventionist group 1, number 1

They found the IVR device quite novel, as some of them had never encountered it before. Initially, when using it, they might have been a bit resistant or worried, wondering what it was. We were there to explain and guide them to use it.

Student interventionist group 2, number 2

Furthermore, student interventionists described their enrollment in the program as eye-opening for themselves, as it enabled them to learn how to apply innovative IVR technology to enrich the lives and improve the well-being of older adults. Some students demonstrated a keen interest in IVR technology and showed a willingness to prepare themselves for future service provision to older adults by participating in this project.

I joined this program because I genuinely enjoy serving older adults, and I found it quite interesting and novel to teach them how to play IVR.

Student interventionist group 2, number 1

I learned how to communicate with older adults, especially since some of them live in nursing homes and rarely have anyone to talk to. We chatted with them and showed care.

Student interventionist group 2, number 2

### Joyful Adventure

The interviews were filled with laughter as the older adults fondly recounted their experiences with the interactive IVRTP. They particularly relished the moments when animals and landscapes seemed to materialize before their eyes, enabling them to explore distant places while remaining indoors. The participants also expressed delight in witnessing the transformation of cities and buildings over the years, marveling at how modern advancements in construction and technology have reshaped urban landscapes. They appreciated the voice introductions provided by the IVRTP, which explained city development, offered tourism guidance on natural landscapes, and introduced various animals. Overall, the older adults expressed a genuine love and appreciation for the IVR elements, including the natural scenery, wildlife, and cities from other countries or regions. Their feelings of happiness and excitement were conveyed through both verbal remarks and nonverbal expressions, such as laughter and smiles.

There have been many large animals. I don’t speak very well. Seeing many places makes me even happier. I can see so many places without having to go there.

Group 7, number 6

Being stuck in the same place every day, seeing faraway places really opens up your view. It makes you feel more open-minded and cheerful.

Group 6, number 4

Student interventionists noted their observations of the positive emotions experienced by older adults during their participation in the IVRTP. They described how older adults felt revitalized when encountering diverse landscapes and animals, which provided a welcome respite from their daily routines.

Throughout the process, we observed that the elderly consistently showed improvements in their emotions. They were very engaged in each IVR environment, which made them quite happy.

Student interventionist group 2, number 1

### Reminiscing Memories

Older adults remarked that the IVRTP enabled them to reminisce about past experiences and memories. The sightseeing locations or cultural events (eg, Chinese-style wedding) evoked old memories, as they had visited or lived in these places or attended such events before. Some were surprised by changes like building and street renovations and eagerly shared stories of their previous visits or residences. Additionally, these memories facilitated peer dialogue, enabling older adults to share and gain a better understanding of each other’s life stories.

With this IVR, even though we haven’t actually been there, it reminds me of places we used to go. I’ve been to places like the Peak before, but now, even if you want to go, you can’t. Still, using this, it’s like...bringing back memories.

Group 5, number 5

Student interventionists observed that the IVRTP facilitates reminiscence among older adults, enabling them to recall and share their life stories. This not only brings immediate joy through the use of IVR but also fosters enhanced peer networking in their daily lives. The multiplayer mode further enriches this experience by allowing residents to share these narratives and life experiences as the IVR scenarios change, all within the context of the residential home. Students reported that older adults displayed increased cheerfulness, breaking away from the monotony of residential home life when they engaged in these IVR scenarios and the associated exchanges of life stories with one another.

So, when I showed them some IVR scenery, like Macau city, a few of the elderly were like, “Wow, I used to live in Macau!” and others were like, “I’m actually Portuguese!” and started speaking Portuguese. It was amazing because they didn’t even know that the person next to them had lived in Macau. IVR program helps them share their past experiences and discover common topics with each other, which is great.

Student interventionist group 1, number 1

### Immersive Escape: a Realistic Journey

Older adults remarked on the immersive features of the IVR program, noting that it provided a realistic journey that went beyond merely viewing scenes to actually experiencing them. They explained that the goggles brought the scenes very close, making them feel as if they were right next to the objects. Additionally, they shared that the sense of realism was enhanced by the IVR’s capability, which allows them to look around and revisit scenes repeatedly, simulating the experience of traveling.

Looking at IVR, it’s like you’re visiting the place yourself, not just seeing the surface scenery on a phone. It’s about experiencing it personally, seeing all these things, and I feel like it’s as if I’ve traveled there myself. It gives that kind of feeling.

Group 3, number 3

I feel the same, haha, exactly. When I see it, it feels really real. Because you get so close, you can see the animals and the mountain tops, and everything feels authentic.

Group 3, number 1

Two participants shared different opinions about the authenticity of the IVR scene:

Yeah, if you’re talking about seeing those animals, everything looks fake. Well, it all comes down to the photographer’s skills, in my opinion.

Group 5, number 5

Well, you could say some things look real and some things...not so much.

Group 5, number 1

Student interventionists also commented on the unique feature of IVR that enables older adults to interact with the scenario and have realistic experiences.

There was this one elderly lady, and when we were “visiting” modern Macau, it was like she was riding in a car. She really got into it, pretending to drive a stick shift and changing gears. We saw her and thought, “Wow, it’s like she’s really driving!”

Group 1, number 1

### Minor Discomforts

Two participants reported experiencing headaches, and one participant mentioned vestibular disturbances while using IVR. To manage these symptoms, they adjusted by reducing the amount of looking around and partially closing their eyes.

When I put the glasses on, I get a headache, so I don’t really feel like looking around. However, some places have undeniably changed completely. When you reach the peak, it’s already very different. The streets all seem quite novel and new.

Group 6, number 2

But I have an inner ear imbalance, so sometimes when things turn, I squint my eyes and don’t dare to look too much, because it makes me feel dizzy. The scene is like 360 degrees, and you can see everything no matter where you turn.

Group 3, number 3

The student interventionists noted similar discomfort experienced by older adults during the intervention. They observed that older adults often tend to endure discomfort without reporting it. This highlights the necessity for careful observation and assessment to ensure that any discomfort is identified and addressed promptly.

Through this program, I’ve learned even more, like how some elderly people won’t express themselves when they’re uncomfortable. They might just feel dizzy and think it’s normal. So, you have to occasionally check in with them, asking, “Hey, are you feeling okay? Do you feel dizzy?” This way, they might admit, “Actually, I do feel a bit dizzy, I don’t want to wear it anymore.” It’s been a learning experience for me.

Group 1, number 1

## Discussion

### Principal Findings

To our knowledge, this is the first reported qualitative study to explore older adults’ as well as student interventionists’ experiences of a structured 6-week IVRTP that integrates tourism, natural scenes, and reminiscence within residential care settings for people with physical disabilities. Interviewing student interventionists can deepen this understanding, elucidate the intervention’s educational value, and inform strategies to sustain the translation of research into service delivery within school practicum curricula. Participants described IVRTP as broadening their horizons by enabling engagement with new technology, learning opportunities, and novel activities. They emphasized the realism of the experience, noting that it afforded an immersive sense of “escape” from the residential home environment and from mobility constraints. The intervention frequently evoked autobiographical memories: participants reflected on past experiences and remarked on changes in familiar tourist destinations over time. Interviews were characterized by laughter when recalling different IVR scenarios; the prevailing positive perspectives may reflect the limited recreational opportunities available to this cohort. These qualitative findings corroborate the quantitative outcomes of our multicenter RCT and extend understanding in two ways: (1) older adults expressed a strong appetite for learning novel activities and acquiring new knowledge and (2) participation elicited reminiscence and prompted the sharing of past life stories among residents. Students’ meaning-making and colearning with older adults underscored the pedagogical value of authentic, community-based learning and intergenerational engagement, reinforcing the integration of a community practicum within the curriculum.

Our findings identified a prominent impact of IVRTP that is underexplored in existing IVR-based studies among older adults—openness and curiosity toward engaging in novel activities and learning [11]. The literature documents age-related declines in curiosity and exploratory behavior, while also indicating that higher levels of curiosity are associated with protection against age-related cognitive decline [46]. Mechanistically, greater curiosity may foster increased engagement with cognitively stimulating environments and experiences, thereby contributing to the development of cognitive reserve [47]. In this study, participants’ appreciation of and excitement about advanced technology and the virtual travel experiences it enabled (eg, visiting unfamiliar places, viewing contemporary versions of familiar locales, and virtually revisiting their hometowns as they appeared decades ago) are described as stimulating their innate curiosity. Unlike a prior IVR study in which older adults’ self-reported quantitative data and qualitative interviews pointed to disease-related knowledge acquisition via interaction with virtual scenarios [48], the present study foregrounds the IVR experience itself.

The findings revealed joy, excitement, and the reliving of positive memories during IVRTP participation, which may help explain and corroborate the significant improvements in psychological well-being measured quantitatively in the full RCT [25]. These results align with existing literature in which participants reported delight and engagement arising from perceived proximity to animals and natural landscapes [7]—an experience associated with increased comfort and reduced heart rate [49,50]. The findings also mirror other IVR-based studies, wherein older adults were reminded of their past and relived happy moments [51], a process linked to fostering positive affect and psychosocial adaptation [52]. Moreover, this aligns with the literature indicating that when direct contact with nature or travel is not feasible among this population, engaging with “virtual nature” yields therapeutic effects; IVR can deepen and materialize specific experiences, promoting older adults’ engagement and acting as a “coenzyme” in the therapeutic process [7].

Findings from the student interventionists reinforce a well-documented benefit of undergraduate experiential learning in health professions: practice embedded in health care settings strengthens learning by connecting theory to authentic contexts and fostering professional identity [53]. Consistent with prior studies of undergraduate nursing and allied health students engaged in placements with purposeful integration of technology [54], student interventionists reported gains in understanding older adults, who turned out to be interested in high technology. In their narratives, students described how participation in IVRTP influenced older adults’ emotions, broke their rigid routines, and stimulated happy memories and stories to share among residents. Such reflection and transformation (ie, change-making in older people’s care) stimulated inspiration about technology-based ways to provide care [55].

Participants reported minor adverse events associated with IVRTP participation; although minor, these events still carry substantial value for research and practice. Student interventionists’ observations mirrored this issue and further indicated that older adults often tolerate and hide discomfort rather than seeking help. Aligned with findings from other IVR-based psychological interventions, cybersickness is typically mild or manageable, underscoring favorable usability and acceptability [11]. Using seated configurations and teleportation-based locomotion [56], alongside recent enhancements in display latency, refresh rates, optical systems, and tracking accuracy [57], may substantially reduce cybersickness. However, given that IVRTP was applied among older adults with physical disabilities—and may be extended to other patient populations in the future—careful monitoring and supportive/comfort measures should be in place.

### Implications

The participants’ and interventionists’ shared experiences are highly informative and carry several practice implications that warrant consideration. The findings indicate that IVR is acceptable, engaging, and feasible for older adults with physical disabilities in residential care when standby usability and technical support are in place, supporting its integration as a structured recreational and therapeutic service in contexts where volunteers or students are available to provide such support. To foster positive affect while avoiding adverse emotions, such as anger at substituting real-world experiences with IVR or fear elicited by high-elevation viewpoints [58], IVR content should be developed under a therapeutically oriented protocol with explicit goals (eg, mood enhancement, reminiscence, and stress reduction), careful curation to avoid common triggers (eg, heights, rapid motion, and crowding), and cultural and personal relevance [59-61]. Implementation should be guided by standardized protocols covering screening for contraindications, informed consent, session parameters (duration, pacing, seated positioning, and supervision), real-time monitoring with clear stop-signal procedures, and postsession debriefing and documentation. Student interventionists can deliver IVR effectively with training in therapeutic communication, older people’s care, device setup and hygiene, troubleshooting, and safety (falls, transfers, and infection control), supported by ongoing supervision and reflective practice. Person-centered adaptation, including accessibility features and alternatives (eg, screens/tablets for those who cannot tolerate headsets), promotes equity and reduces exclusion. Reliable hardware, hygiene protocols, appropriate spaces (quiet and comfortable seating with head/neck support), and clear risk management (acclimatization, criteria for pausing/terminating, and escalation pathways) are essential.

Furthermore, implications for future research are noteworthy. Studies should broaden outcome assessment to include openness to experience, curiosity for learning, and reminiscence, alongside other psychological parameters such as stress and quality of life, using designs to capture mechanisms and effectiveness. Implementation-focused studies should engage stakeholders beyond students and residents to include family members, clinicians, administrators, information technology engineers and facilities staff, and vendors through co-design and implementation-science frameworks to identify and address barriers related to cost, technical literacy, workflow integration, staffing, and infrastructure, including economic evaluations and training/fidelity models. Future studies may explore usability and experiences with interactive features in this cohort, as participants in the present study relied primarily on interventionists to operate the IVR devices.

### Limitations

This study has several limitations. First, participants were interviewed retrospectively to describe their experiences with the IVRTP, which introduces recall bias and may lead to over- or underreporting of experiences. Second, caution is warranted when interpreting these findings, as the perspectives of participants who withdrew from the study and discontinued data collection due to IVR sickness were not captured. Specifically, among the 133 participants allocated to the intervention group, seven withdrew from the intervention for this reason, raising the possibility that the findings may be disproportionately positive. Third, the use of focus groups carries inherent constraints, including group dynamics that can suppress dissenting views (eg, conformity and groupthink), the dominance of more vocal participants, and reduced willingness to disclose sensitive or negative experiences in a shared setting. Lastly, this study did not require participants to have any particular educational background or level of digital literacy, and student interventionists were on standby to provide immediate technical guidance and support, resulting in limited information regarding IVR usability.

### Conclusion

IVRTP is perceived as an accessible, enjoyable, and beneficial adjunct to routine activities, offering meaningful and eye-opening activities for older adults with physical disabilities. Participants reported that it broadened their horizons by introducing new technologies and facilitating learning, with vividly realistic experiences that provided an immersive respite from residential care environments and mobility constraints. IVRTP also evoked reminiscence and facilitated reflection on life events, and interviews were marked by laughter and affirmations of positive emotion. These intergenerational encounters similarly highlighted students’ meaning-making alongside older adults, underscoring the pedagogical value of authentic, community-based engagement and supporting the integration of a community practicum within the curriculum.

## Data Availability

The datasets generated or analyzed during this study are not publicly available due to ethical concerns but are available from the corresponding author upon reasonable request.

## Appendix

Multimedia Appendix 1 SRQR checklist.

Multimedia Appendix 2 Interview guide.

## Abbreviations

IVR: immersive virtual reality
IVRTP: Immersive Virtual Reality Therapeutic Programme
LTCF: long-term care facilities
MBI: modified Barthel Index
PPIE: patient and public involvement and engagement
