# Supplementary material for: Experiences of Immersive Virtual Reality Among Older Adults With Physical Disabilities: Qualitative Process Evaluation of a Multicenter Randomized Controlled Trial
Source: J Med Internet Res. 2026 Oct 29;28:e101815. doi: 10.2196/101815 (PMC13760328; doi:10.2196/101815)
Supplement: Supplementary file 2 [file jmir_v28i1e101815_app2.docx]

**Interview guide**

a. Older adults’ version

1. What motivated you to take part in this immersive virtual reality activity?

2. How would you describe your experience of participating?

3. Which parts of the activity were most surprising or memorable to you?

Probe: After completing the IVR sessions, did your views of IVR change in any way? If so, how?

4. In what ways, if at all, did this activity affect your mental health? Could you tell me more?

Explanation for participants: By “mental health,” we mean things like whether you feel happy, able to relax, or not lonely, etc.

5. What are your views on using IVR within residential care homes?

6. If a similar activity were offered in the future, how would you feel about taking part? How, if at all, would you recommend it to family or friends?

7. What aspects of the IVR program or its arrangements do you think could be improved?

8. Do you have any other comments or suggestions about the program?

b. Student interventionists’ version

1. What motivated you to participate in the immersive virtual reality therapeutic program?

2. How would you describe your experience as interventionists?

3. In what ways, and to what extent, did this program influence your competence to help older adults use digital technologies?

Prob: Can you share specific examples that made you feel you could support older adults more effectively in using digital technologies?

4. What was the most impressive thing you learned when working with older adults?

Prob: Before participating, how much did you understand about older adults’ needs and age-related barriers (e.g., hearing impairment)?

5. To what extent did the program shape or influence your views of older adults?

6. After completing the program, what do you consider your biggest takeaway? How did the experience align with your expectations before you joined?

7. What aspects of the program or its arrangements do you think could be improved? (e.g., course length, content, group size)

8. Do you have any other comments or suggestions about the program?
